# Epidemiology of Keratoconus in India: A Systematic Review and Meta-Analysis of Indian Study Populations

**DOI:** 10.3390/vision10020020

**Published:** 2026-04-09

**Authors:** Matteo Ripa, Chiara Schipa, Paola Aceto, Sushad Prasad, Neeraj Apoorva Shah

**Affiliations:** 1 Department of Ophthalmology, Sankara Eye Hospital, Jaipur 302039, Rajasthan, India; 2 Catholic University “Sacro Cuore”, 00168 Rome, Italy; 3 Department of Emergency, Anesthesiological and Reanimation Sciences, Fondazione Policlinico Universitario A. Gemelli IRCCS, 00168 Rome, Italy; 4 Department of Ophthalmology, Sankara Eye Hospital, Krishnankoil 626190, Tamil Nadu, India

**Keywords:** keratoconus, India, prevalence, systematic review and meta-analysis, incidence

## Abstract

(1) Background: To synthesize available evidence on the prevalence of keratoconus (KC) reported in Indian study populations and describe its demographic distribution. (2) Methods: PubMed, Embase, and Scopus were checked using free text and controlled vocabulary. A random-effect meta-analysis of pooled prevalence and its 95% confidence intervals (CIs) for KC among study participants recruited in India was conducted using exact binomial distributions and the Freeman–Tukey double-arcsine transformation. To identify potential sources of variability, we conducted subgroup analyses by dividing the data by geographic region, KC assessment, and study population. The methodological quality of each study was assessed using the Newcastle–Ottawa scale (NOS). Evidence quality was evaluated using the GRADE system. (3) Results: Across included studies, the total number of KC cases was 16,164, and sample sizes ranged from 152 to 2,384,523 participants. Prevalence estimates varied markedly across studies, reflecting substantial heterogeneity in study design, diagnostic criteria, and population characteristics. Most studies were conducted in high-risk clinical settings, limiting generalizability to the general population. Subgroup analyses showed no significant differences by geographic region or diagnostic modality (*p* = 0.79 and 0.07, respectively). There was a statistically significant subgroup effect (*p* < 0.001) in the study population. The reported prevalence among females ranged from 0.00 to 0.04, while the pooled prevalence estimate was 0.02 (95% CI: 0.00–0.04). Four cross-sectional studies scored 8–10 on the NOS. (4) Conclusions: Our meta-analysis synthesized the currently available evidence on keratoconus prevalence across Indian study populations, highlighting substantial variability across studies and emphasizing that estimates should be interpreted within their specific study contexts rather than as representative of the national population.

## 1. Introduction

Keratoconus (KC) is a non-inflammatory bilateral and asymmetric corneal disease characterized by progressive thinning and steepening of the cornea [1]. It leads to irregular astigmatism and impaired visual acuity and still represents the most common cause of corneal transplant in younger non-Caucasian patients [2]. KC affects patients in their adolescence and tends to progress until the fourth decade of life [3], thus impacting patients’ quality of life and mental health, being also associated with depressive disorders and psychosis [4,5].

Despite KC affecting several patients worldwide, there is high variability in demographic data and morbidity measures. Indeed, despite a recent meta-analysis reporting a KC prevalence of 1.38 per 1000 people in the whole population [6], KC prevalence ranges from 0.5 to 2.3 per 1000 people in the Western countries and up to 40 per 1000 in the rural areas of Iran with Caucasian populations having a KC prevalence rating of under 1000 per 100,000 persons, and Asian and Middle Eastern populations having ratings between 1500 and 5000 per 100,000 persons [7]. In 2020, Akowua et al. were the first to analyze the epidemiology of KC in Africa, reporting an overall KC prevalence of 7.9% (95% CI: 2.5–16.0%) [8]. According to these data, high variability in prevalence might be related to differences in geographic locations and patient cohorts.

India is considered a high-burden region for KC, although epidemiological estimates vary widely. Nonetheless, only a few cross-sectional studies have investigated KC prevalence in India [9,10,11,12,13,14,15,16,17,18]. In 2009, Jonas et al. reported a KC prevalence of 2.3% among Indians aged 30 [13]. Recently, Das et al., in their cross-sectional hospital-based study, including data from 2,384,523 patients, found 14,749 patients with KC in at least one eye, reporting an overall prevalence of 0.62% [95% confidence interval (CI): ±0.0062%] [17]. To the best of our knowledge, no systematic reviews or meta-analyses have been conducted to evaluate morbidity measures and the demographic distribution of KC in India. Therefore, we aimed to synthesize available evidence on the prevalence of keratoconus (KC) reported in Indian study populations and describe its demographic distribution.

## 2. Materials and Methods

### 2.1. Search Strategy

To investigate the epidemiology of KC in India, we searched three online databases (PubMed, Embase, and Scopus) from inception until 8 June 2025, using both free text and controlled vocabulary (MeSH or Emtree). The study’s impact on patient, intervention, comparison, and outcome (PICO) was used to define the search strategy: population (pediatric and adult populations in India), intervention (none), comparison (none), and outcome (prevalence of KC). The search approach integrated controlled vocabulary with keywords as outlined in the prior literature. PubMed article searches used the Medical Subject Headings (MeSHs) controlled vocabulary, whereas the Embase Subject Headings (EMTREE) controlled vocabulary was employed in the EMBASE. In addition, Boolean operators such as “AND” and “OR” were employed to extend and narrow the research. Additionally, the study was performed without database filters to secure a larger sample and minimize potential loss. To include all studies performed in India exploring the KC prevalence, we comprised the following terms: “India” and “Name of each of the 28 States of India” AND “Keratoconus.” To ensure thoroughness, we considered individual words and medical terminology that might substitute for morbidity measure, such as “Prevalence,” “Frequency,” and “Incidence.” Finally, we hand-searched the reference lists of included publications to identify additional research that was not found during the initial database search. This proceeded until adding more terms yielded no new results.

The detailed search strategy and PRISMA Checklist are reported in Supplementary Methods S1 and S2.

### 2.2. Study Selection, Data Extraction, and Data Synthesis

This review was conducted following the PRISMA guidelines. The research has been registered in PROSPERO (identifier CRD42024557328). We included all studies, regardless of the patient’s age, that reported data regarding the epidemiology of KC in India. Reports that offered information to compute the prevalence of KC were included, even if the prevalence was not explicitly documented. In that case, the prevalence was estimated by dividing the number of people with KC by the total number of study participants.

KC was classified as a corneal ectatic disease identified with progressive cone-shaped steepening of the cornea and diagnosed relying on slit-lamp biomicroscopic (e.g., Vogt’s striae, Fleischer’s ring, Rizzuti sign, and stromal thinning) and topographic/tomographic features suggestive of KC, and/or corneal distortion in either eye (as seen with keratometry or retinoscopy). Articles were excluded if they were unavailable in English and did not offer a clear description of morbidity measures to provide a measure of the extent of KC in the population. Furthermore, our study excluded literature review studies, theses and dissertations, book chapters, technical reports, and publisher correspondence. Two investigators (M.R. and C.S.) independently retrieved baseline and outcome data. Two co-authors (P.A. and N.A.S.) were contacted to decide if an agreement could not be reached. The reasons for exclusion were recorded. We extracted the following data from each article: first author, year, state, study design, study population, age range, total sample size, number of patients with KC, number of eyes with KC, age of KC patients, gender, site, diagnostic test used, risk factors and/or ocular co-morbidities, KC stage, KC definition. Although meta-regression was planned in the PROSPERO protocol, it was not performed due to limited study-level data, while post hoc subgroup analyses by gender and laterality were added based on data availability and clinical relevance. Covidence systematic review software© (Veritas Health Innovation, Melbourne, Australia) was employed to record and evaluate the study data between 8 May 2024 and 8 June 2025 [19].

### 2.3. Risk of Bias Assessment

The methodological quality of each study was independently assessed by two authors (M.R. and C.S.) using the Newcastle–Ottawa Scale (NOS) [20]. Each reviewer’s unique appraisal of the quality assessment data was then compared. If no agreement could be reached, M.R. and C.S. discussed the disagreements for adjudication with a third author (N.A.S.), who reviewed potential conflicts in adjudication if a consensus could not be reached.

### 2.4. Assessment of Quality of Evidence

The quality of evidence for each outcome was evaluated using the Grading of Recommendations Assessment, Development, and Evaluation (GRADE) profiler version 3.6 in conjunction with the GRADE system consensus of two authors (M.R. and C.S.). Studies start with a high-quality rating; however, this rating may decrease due to various factors, including risk of bias, inconsistency, indirectness, imprecision, and publication bias. This system distinguishes the following four quality categories for the evidence: high, moderate, low, and extremely low [21,22].

### 2.5. Statistical Analysis

We performed a random-effect proportional meta-analysis to pool study-level keratoconus prevalence estimates from studies conducted in India (i.e., across the included Indian study populations). Because recruitment settings and diagnostic criteria differed substantially, pooled estimates were interpreted as summaries across study contexts rather than national population prevalence. The random-effect proportional meta-analysis was based on exact binomial distributions, which compared the number of “events” to the number of “non-events” in a sample. The analysis utilized Freeman–Tukey double-arcsine transformation via the “metaprop” command in STATA (StataCorp LLC, College Station, TX, USA), version 18.0. A random effects model was employed because of the expected high levels of heterogeneity in the prevalence of KC in India. The outcomes of interest, including the prevalence of KC and the prevalence of KC according to gender and laterality, were calculated and reported with a 95% CI. We significantly assessed between-study heterogeneity when the *p*-value for the Q-test was less than 0.10 or the I^2^ statistic was 50% or higher [23]. Additionally, the heterogeneity was examined through a Galbraith plot and sensitivity analysis employing the leave-one-out method. The GRADE profiler program was used to capture the GRADE evidence evaluation findings. We conducted subgroup analyses to identify potential causes of variability by stratifying the data based on geographic region (North vs. East vs. West vs. South), KC assessment (corneal topography vs. keratometry vs. not reported), and study population (allergic eye disease vs. vernal keratoconjunctivitis vs. study of refractive surgery candidates vs. contact lenses wearers vs. general population). Barker et al. suggest that a high I^2^ in proportional meta-analysis does not inherently indicate data inconsistency, and the outcomes of this test should be approached with caution. Egger’s test and funnel plots have been developed in the context of comparative data, and there is no evidence that proportional data adequately adjust for these tests. According to Barker et al., even though these tests could be used in the proportional meta-analysis, there is little evidence that proportional data appropriately account for them. Furthermore, the idea that positive findings are more frequently reported is not always true for proportional research, as there is no clear definition or consensus on what constitutes a positive result in a meta-analysis of proportion. As a result, as previously reported by Barker and his collaborators, we did not adopt them for our proportionate meta-analyses, and publication bias was instead qualitatively evaluated [24]. Statistical significance was established with a two-sided *p*-value of 0.05.

## 3. Results

### 3.1. Study Selection

**Figure 1:**
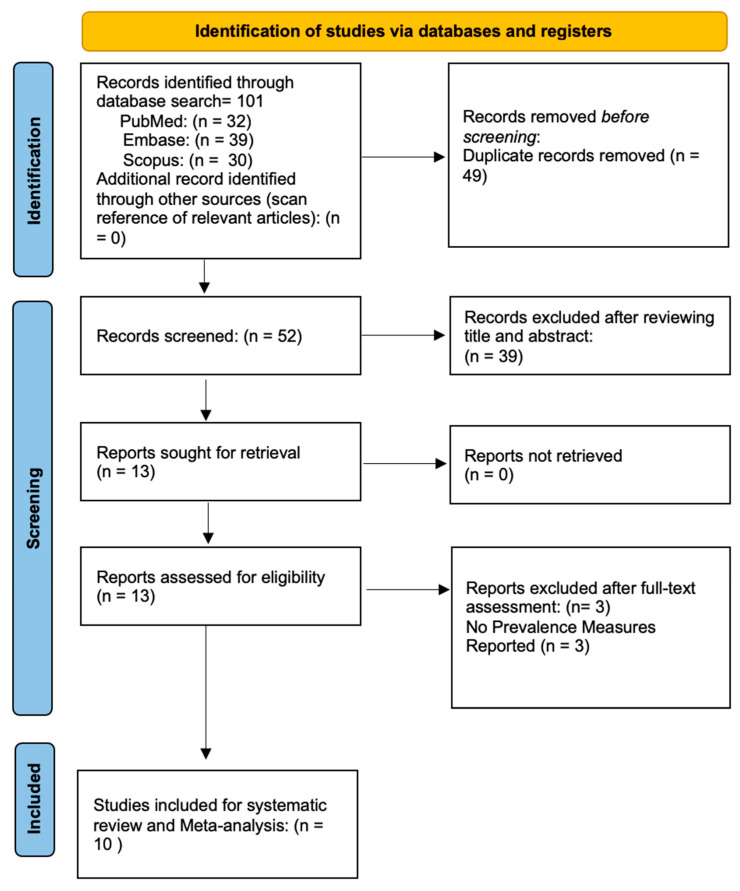
Flow diagram of the study selection process.

Figure 1 illustrates the flow chart outlining our analytical selection screening process. The preliminary search resulted in 101 indexed articles (32 from PubMed, 39 from Embase, and 30 from Scopus). Further searching the retrieved studies’ references list yielded no additional research. After duplication removal, we screened 49 articles. After assessing the titles and abstracts, we excluded 39 articles, and only 13 full-text studies were retrieved and assessed for final eligibility. Furthermore, three articles were excluded because neither the morbidity measures nor the information required to compute the prevalence were retrieved, resulting in ten studies in the systematic review and the meta-analysis [9,10,11,12,13,14,15,16,17,18].

### 3.2. Study Characteristics

Table S1, available in the Supplementary Material S3, summarizes the primary studies’ characteristics. We assessed ten cross-sectional studies [9,10,11,12,13,14,15,16,17,18]. The number of participants ranged from 152 to 2,384,523 [12,17], for a total of 2,448,470 patients, whereas the total number of KC patients ranged from 11 to 14,749, for a total of 16,164 KC patients with a median age ranging from 22.61 ± 8.23 to 53.2 ± 11.3 years [9,10,11,12,13,14,15,16,17,18].

Overall, two studies recruited data from the Northern Indian population settled either in Delhi or Jammu and Kashmir [10,13], five studies from the Southern Indian Population settled in Karnataka or Telangana [9,14,15,16,17], three studies from patients settled in Western India such as Gujarat and Maharashtra [11,12,15,17,18], and two studies retrieved data from patients settled different countries such as Telangana, Andhra Pradesh, Odisha, and Karnataka and was treated as a southern majority population in subgroup analyses [15,17].

### 3.3. Study Population

The study population differed among the included studies. Indeed, out of the ten included cross-sectional, two articles reported the total prevalence of KC by either analyzing the electronic medical records of all patients that attended the hospital during the study period regardless of their ocular co-morbidities and/or sub-ophthalmology specialty referral or data retrieved from patients recruited for screening purposes from rural villages far away from the main tertiary eye center, respectively [17,18]. According to them, the total prevalence of KC in the whole population was 2.3 ± 0.2% and 0.62% (95% CI: ±0.0062%), respectively [9,10,11,12,13,14,15,16,17,18]. Four studies were conducted in VKC patients [9,12,13,14,15], one in AED patients [15], two in patients attending contact lens clinics [10,16], and one study reported the prevalence of KC in patients presenting for refractive surgery [10,11,16].

### 3.4. KC Diagnosis and Grading

The included studies (10 cross-sectional, with 2,448,470 total participants) used different diagnostic tests [9,10,11,12,13,14,15,16,17,18]. Indeed, KC was diagnosed according to topographical and biomicroscopic features in four studies [10,12,14,17]. In contrast, one study relied on keratometry values defining the KC as per any corneal refractive power of more than 48 diopters (Ds) [18]. Five studies did not report the diagnostic procedures previously employed for KC diagnosis [9,11,13,15,16].

While Jonas et al. considered the diagnosis of KC in any patients whose keratometry values were higher than 48 D [18], Umale et al. diagnosed the KC using the modified Rabinowitz–McDonnell test characterized by K value > 47.2 D and/or inferior–superior (I–S) value of >1.4 D [12]. Finally, Das et al. graded the KC severity by employing the Amsler–Krumaich classification [17], whereas Fatima et al. employed the keratometry values identifying a mild KC if both meridians’ curvature was less than 45 D and severe KC if one or both meridians curvature measured more than >62 D [10].

### 3.5. Meta-Analysis and Subgroup Meta-Analyses of KC Prevalence

A proportional random-effect meta-analysis was conducted to pool keratoconus prevalence estimates from studies conducted in India. Across included studies, the number of keratoconus cases was 16,164, and sample sizes ranged from 152 to 2,384,523 participants [9,10,11,12,13,14,15,16,17,18]. Prevalence estimates were pooled from ten studies, which were the only investigations reporting sufficient data to compute keratoconus prevalence [9,10,11,12,13,14,15,16,17,18].

**Figure 2:**
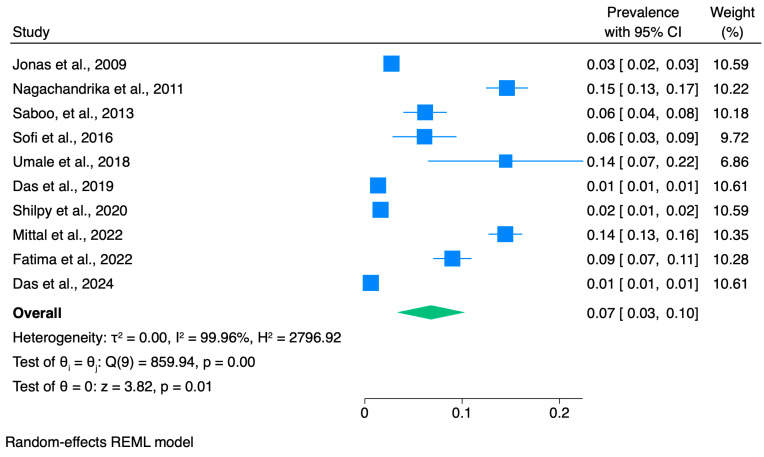
Random-effect meta-analyses of the keratoconus prevalence in India. The diamond represents the pooled estimated prevalence; box sizes reflect the relative weight apportioned to studies in the meta-analysis [9,10,11,12,13,14,15,16,17,18].

The pooled prevalence of KC across studies conducted in India was equal to 0.07 (7%), with a CI between 0.03 and 0.10 (I^2^ = 99.96, z: 3.82, and *p* = 0.01). However, most included studies were conducted in selected high-risk clinical populations (e.g., VKC/allergic eye disease, refractive surgery candidates, and contact lens clinic attendees) rather than in representative general population samples. Therefore, this pooled estimate should not be interpreted as a national population prevalence for India, but as a summary measure across heterogeneous study settings (Figure 2).

Our meta-analysis found considerable heterogeneity among the studies [9,10,11,12,13,14,15,16,17,18]. We conducted a sensitivity analysis to identify outliers and assess the impact of each individual study on the stability of the pooled data by excluding one study at a time, using a “leave-one-out meta-analysis” approach (Figures S1 and S2, available in the Supplementary Material S4). The findings revealed that no significant changes occurred in the pooled data when any single study was excluded. Additionally, the Galbraith plot used for heterogeneity analysis indicated consistency across studies. Therefore, the results of the sensitivity analysis confirmed that our pooled data were statistically stable and reliable.

**Figure 3:**
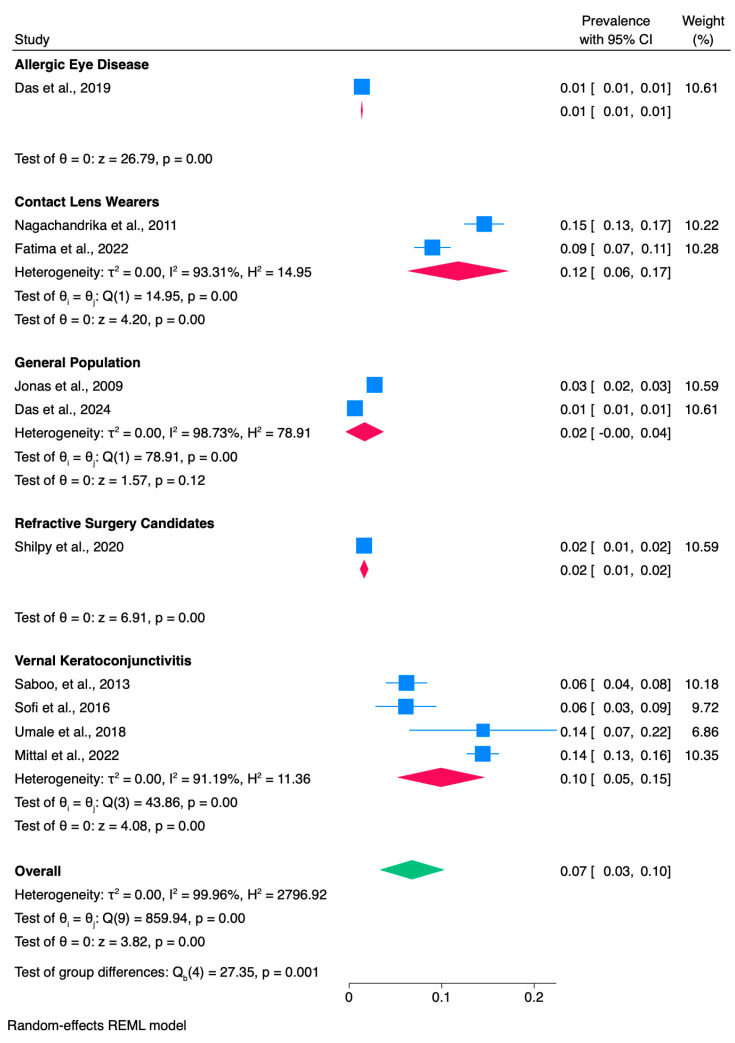
Subgroup analysis of the keratoconus prevalence in India according to study population (allergic eye disease vs. vernal keratoconjunctivitis vs. study of refractive surgery candidates vs. contact lens wearers, general population). The diamond represents the pooled estimated prevalence for each meta-analysis; box sizes reflect the relative weight apportioned to studies in the meta-analyses [9,10,11,12,13,14,15,16,17,18].

Despite the meaningful high heterogeneity among the studies, we conducted a subgroup analysis. Subgroup analyses stratified by categorical study-level characteristics are reported in Figures S3 and S4, available in the Supplementary Material S4, and Figure 3.

These characteristics include geographic region (North vs. East vs. West vs. South), KC assessment (corneal topography vs. keratometry vs. not reported), and study population (allergic eye disease vs. vernal keratoconjunctivitis vs. study of refractive surgery candidates vs. CL wearers, general population). The test for differences within subgroups shows no statistically significant effects in KC assessment and geographic region (*p* = 0.07, *p* = 0.79, respectively) (Table S2, available in the Supplementary Material S3). Furthermore, it indicates a statistically significant subgroup effect (*p* = 0.001) in the study population, suggesting that this variable has a statistically significant impact on KC prevalence in India. However, there is substantial unexplained heterogeneity among the studies, and only one study reported the KC prevalence in the allergic eye disease (AED) and refractive surgery candidates, respectively, limiting the strength of conclusions for these subgroups.

Only five studies reported the prevalence of KC and the patients’ gender [10,11,12,17,18]. Das et al. in 2024 found that the prevalence of KC was significantly greater in males compared to females (*p* < 0.001) [17], whereas Jonas et al. in 2009 reported a significantly higher prevalence in females (*p* = 0.001) [18]. The other studies did not report any inferential statistics related to gender [10,11,12]. Therefore, the overall prevalence among males and females in India was pooled from five studies. The prevalence of KC in the included studies was 0.05 (5%) (95% CI: 0.01–0.09, z = 2.20; I^2^ = 99.75%, *p* = 0.03) across all subjects, regardless of gender.

The pooled results showed no statistically significant prevalence in KC male patients. The range of reported prevalence among males was from less than 0.01 to 0.11, while the pooled prevalence estimate among males was 0.03 (3%) (95% CI: 0.00–0.06, z: 1.77; I^2^ = 99.84%, *p* = 0.08). The range of reported prevalence among females was from less than 0.01 to 0.04, while the pooled prevalence estimate among females was 0.02 (2%) (95% CI: 0.00–0.04, z: 2.56; I^2^ = 89.49%, *p* = 0.01). Figure S5, available in the Supplementary Material S4, shows the forest plot for KC prevalence in the included studies in males and females.

Only two studies reported the prevalence of KC according to laterality [12,17]. Das et al. in 2024 found that KC was bilateral and unilateral in 12,954 (87.83%) and 1795 (12.17%) cases, respectively [17], whereas Umale et al. found 6 out of 11 (54.54%) patients with bilateral KC and 5 out of 11 (45.45%) with unilateral disease [12]. Therefore, the overall prevalence of KC according to laterality in India was pooled from two studies. The pooled results showed no statistically significant prevalence in KC patients according to the laterality (*p* = 0.31). The prevalence of KC in the included studies was 0.07 (7%) (95% CI: 0.07–0.20, z: 0.98; I^2^ = 91.52%, *p* = 0.31) in all subjects. The range of reported prevalence in monolateral KC was from 0.03 to 0.09, while the pooled prevalence estimate among males was 0.03 (3%) (95% CI: 0.03–0.09, z: 0.98; I^2^ = 77.80%, *p* = 0.33). The range of reported prevalence in bilateral KC was from 0.04 to 0.11, while the pooled prevalence estimate among males was 0.03 (3%) (95% CI: 0.04–0.11, z: 0.87; I^2^ = 84.35%, *p* = 0.38). Figure S6, available in Supplementary Material S4, shows the forest plot for KC prevalence in the two studies and monoliteral and bilateral KC.

### 3.6. Assessment of Methodological Quality and Risk of Bias

Table S3, available in the Supplementary Material S3, summarizes the risk of bias evaluation of all studies. Overall, four cross-sectional studies reached a total score between 8 and 10 [12,15,17,18], while five reached a total score of 6 [9,10,11,13,14] and one reached a score of 7 [16]. Indeed, five studies did not report the diagnostic procedures previously employed for KC diagnosis [9,11,13,15,16] and only four studies evaluated the potential confounders by either subgroup analysis or multivariable analysis [12,13,17,18]. The quality of evidence for our primary outcome was very low according to the GRADE methodology (Table S4, available in the Supplementary Material S3).

## 4. Discussion

Our systematic review and meta-analysis synthesize KC prevalence data from studies conducted in India across heterogeneous recruitment settings. Prevalence estimates varied markedly and were characterized by extreme between-study heterogeneity (I^2^ = 99.96%), largely reflecting differences in study population (general population vs. high-risk clinical cohorts) and diagnostic criteria. Accordingly, the pooled estimate should be interpreted as a summary across the included study contexts rather than as a national population prevalence for India, because most of the contributing studies were based on selected high-risk clinical cohorts and not on representative population-based samples [9,10,11,12,13,14,15,16,17,18].

Multiple studies and numerous meta-analyses conducted globally support this evidence, suggesting a higher prevalence of KC in Eastern countries. Nonetheless, although our results were similar to those of Akowua et al., who analyzed KC prevalence in Africa and found it higher than that reported in studies conducted in Western countries, we observed substantial heterogeneity in our meta-analysis. Several reasons and factors can explain the high heterogeneity and variability among the recruited studies. Indeed, in our meta-analysis, there were only two population-based studies that evaluated the overall prevalence of KC by either analyzing the electronic medical records of all patients that attended the hospital during the study period, regardless of their ocular co-morbidities and/or sub-ophthalmology specialty referral or data retrieved from patients recruited for screening purposes from rural villages far away from the main tertiary eye center [17,18]. The other eight included studies were conducted in cohorts at high risk of KC or in populations with a high likelihood of identifying KC patients, such as those seeking refractive surgery or those with VKC. To be specific, four studies were conducted in VKC patients [9,12,13,14,15], one in AED patients [15], two in patients attending contact lens clinics [10,16], and one study reported the prevalence of KC in patients presenting for refractive surgery [11]. This distribution aligns with the findings of our subgroup meta-analysis, which showed a lower pooled prevalence of KC in the general population [0.02, CI: (0.00, 0.04), *p* < 0.001, I^2^: 78.91%)] compared to higher estimates in VKC patients [0.10, CI: (0.05, 0.15), *p* < 0.001, I^2^: 91.19%)]. While our data cannot demonstrate a causal association, the observed pattern is consistent with previous studies suggesting a link between VKC and increased KC risk.

The retrieved prevalence of KC in the general population was consistent with the previous findings reported in the literature by Hashemi et al., who found a worldwide prevalence of 1.38 per 1000 population [6]. In addition, KC has been reported as a possible consequence of VKC, possibly due to reiterated eye rubbing and inflammatory mediator-induced tissue degradation [25]. According to Naderan et al., those with VKC had a 7.84 times higher chance of developing KC [26]. Merdler et al. found an OR of six for developing KC in patients with concomitant allergic conjunctivitis, chronic blepharitis, or VKC [27]. Our findings were consistent with those previously reported in Nepal and Brazil. In 2005, Dantas et al. reported a KC prevalence in VKC Brazilian patients of 9.85% when analyzing clinical signs and 22.53% when videokeratography was used [28]. In contrast, Gautam et al. reported that 11.3% of patients with VKC had concomitant KC [29].

The different KC diagnostic criteria used across the studies further increased heterogeneity among the included studies, thereby representing a concerning source of bias. Indeed, KC was diagnosed according to topographical and biomicroscopic features in four studies [10,12,14,17], whereas one study relied on keratometry values defining the KC as per any corneal refractive power of more than 48 D [18]. Five studies did not report the diagnostic procedures previously employed for KC diagnosis [9,11,13,15,16]. Although the test for subgroup differences did not reach statistical significance (*p* = 0.07), our subgroup meta-analysis found a higher prevalence of KC in patients undergoing corneal topography [0.09, CI: (0.03, 0.16), *p* < 0.001, I^2^: 98.24%] [10,12,14,17] compared to those diagnosed with keratometry only [0.03, CI: (0.02, 0.03)] [18]. Indeed, keratometers are less sensitive in detecting corneal diseases than corneal topography, as they measure two points at a 2.25–4 mm zone in the central cornea, providing information regarding the curvature, not the corneal shape. Corneal topography, in contrast, provides multiple corneal maps that represent corneal measurements, allowing the clinician to immediately detect any corneal ectatic disorder [30].

In spite of animal research suggesting a gender-specific KC relationship [31], human epidemiological investigations have revealed the following controversial finding: males have a 2–5 times greater prevalence of KC than females, or females have a higher risk of developing KC [32,33]. Notwithstanding, the prevalence of females with KC was slightly inferior compared to males [0.02, (0.00, 0.04), *p* = 0.01, I^2^: 98.49% vs. 0.03, CI: (0.00, 0.06), *p* = 0.08, I^2^: 99.84%)]. The higher prevalence in men could be attributed to different factors, such as the higher risk of being exposed to environmental factors, such as sunlight exposure, dust, and other allergens that can increase eye rubbing and allergy, thus increasing the risk of KC. Finally, KC may develop earlier and evolve more rapidly in men than in women, which might contribute to its higher prevalence [34].

One of our study’s strengths is that we included all available population-based studies reporting KC prevalence in India, allowing a comprehensive overview of the available epidemiological evidence. Our subgroup analysis meaningfully explained the high heterogeneity related to the different population studies across the included studies. However, this systematic review and meta-analysis present several limitations.

First, we restricted our literature search to studies that explicitly reported prevalence data, as outlined in the search strategy. Second, our research comprises only ten studies. Third, different population targets and different employed KC diagnostic criteria represent a high source of bias. Notably, differences in diagnostic methods across studies, particularly between keratometry and corneal topography, introduce a risk of measurement bias. Specifically, in 50% of the included studies, the diagnostic procedures used to identify keratoconus were not clearly reported. This lack of specification represents a potential source of misclassification bias, as studies using less sensitive or undefined diagnostic approaches may underestimate or inconsistently identify cases compared to those employing standardized methods such as corneal topography or tomography. Consequently, this limitation may have contributed to the substantial heterogeneity observed and reduced the comparability of prevalence estimates across studies.

Fourth, as expected, we observed considerable heterogeneity in all meta-analyses, which was only partially explained by our subgroup meta-analysis, alerting that other unexplored causes of heterogeneity exist and may have heavily biased our results. Additionally, we were unable to perform age standardization due to inconsistent reporting of age across studies, which limits the comparability of prevalence estimates and may further contribute to the observed heterogeneity. Lack of age standardization represents a significant limitation given the known age dependence of KC prevalence. As age ranges varied widely across studies, comparability is severely compromised. Fifth, we included only cross-sectional studies more prone to selection bias than other studies’ designs. Sixth, we should consider that there was a high difference among sample sizes in the recruited studies that could have further biased the results. Seventh, although meta-regression is often considered when a sufficient number of studies is available, its validity depends on the availability of consistent and appropriately defined study-level covariates. In the present analysis, key variables were either inconsistently reported, not uniformly defined, or in some cases only available at the individual level, making them unsuitable for study-level modeling. Under these conditions, performing meta-regression would have violated key methodological assumptions and risked producing unstable or potentially misleading results. Therefore, meta-regression was not performed, and subgroup analyses were considered a more appropriate approach. Finally, the extremely high heterogeneity observed in our meta-analysis (I^2^ = 99.96%) significantly limits the interpretability of the overall pooled prevalence estimate. Because of the wide range of populations included, the combined estimate may lack epidemiological and clinical meaning. Thus, despite subgroup analyses partially addressing this issue, the general pooled prevalence should be interpreted with caution. In conclusion, our meta-analysis synthesized the currently available evidence on keratoconus prevalence across Indian study populations, highlighting the wide variability across studies and the importance of population-specific estimates in interpreting disease burden.

Given the substantial heterogeneity and inclusion of clinically distinct populations, the pooled estimate should be regarded as exploratory rather than definitive. Therefore, the pooled estimate primarily reflects the distribution of keratoconus across heterogeneous, often high-risk clinical contexts and should not be interpreted as a national population prevalence for India.

## Data Availability

No new data were created or analyzed in this study.

## Supplementary Materials

The following supporting information can be downloaded at https://www.mdpi.com/article/10.3390/vision10020020/s1, Supplementary Material S1: detailed search strategy. Supplementary Material S2: PRISMA checklist. Supplementary Material S3: Supplementary tables (Tables S1–S4: study characteristics, subgroup analyses, risk of bias assessment using the Newcastle–Ottawa Scale, and GRADE evaluation). Supplementary Material S4: Supplementary figures (Figures S1–S6: Galbraith plot, leave-one-out analysis, and subgroup meta-analyses by geographic region, keratoconus assessment, gender, and laterality). Reference [35] has been mentioned in the text.

## Abbreviations

The following abbreviations are used in this manuscript:

KC	Keratoconus
CI	Confidence Interval
CL	Contact Lens
EMTREE	Embase Subject Headings
GRADE	Grading of Recommendations Assessment, Development, and Evaluation
AED	Allergic Eye Disease
MeSH	Medical Subject Headings
NOS	Newcastle–Ottawa Scale
PRISMA	Preferred Reporting Items for Systematic Reviews and Meta-Analyses
PROSPERO	International Prospective Register of Systematic Reviews
VKC	Vernal Keratoconjunctivitis
D	Diopters
I–S value	Inferior–Superior Corneal Power Difference
PICO	Patient, Intervention, Comparison, and Outcome

## References

[1] Pedrotti E., Chierego C., Bonacci E., De Gregorio A., De Rossi A., Zuliani A., Fasolo A., Marchini G. (2020). New Treatments for Keratoconus. Int. Ophthalmol..

[2] Sarezky D., Orlin S.E., Pan W., VanderBeek B.L. (2017). Trends in Corneal Transplantation in Keratoconus. Cornea.

[3] Al Zabadi H., Shehadeh M., Amro L., Ghattass N., Taha I. (2023). Vision-Related Quality of Life among Patients with Keratoconus: A Cross Sectional Study. Front. Med..

[4] Panthier C., Moran S., Bourges J.L. (2020). Evaluation of Vision-Related Quality of Life in Keratoconus Patients, and Associated Impact of Keratoconus Severity Indicators. Graefes Arch. Clin. Exp. Ophthalmol..

[5] Alfardan F., Alsanad M.H., Altoub H.A. (2023). Prevalence of Psychiatric Illness Among Keratoconus Patients. Cureus.

[6] Hashemi H., Heydarian S., Hooshmand E., Saatchi M., Yekta A., Aghamirsalim M., Valadkhan M., Mortazavi M., Hashemi A., Khabazkhoob M. (2020). The Prevalence and Risk Factors for Keratoconus: A Systematic Review and Meta-Analysis. Cornea.

[7] Pearson A.R., Soneji B., Sarvananthan N., Sandford-Smith J.H. (2000). Does Ethnic Origin Influence the Incidence or Severity of Keratoconus?. Eye.

[8] Akowuah P.K., Kobia-Acquah E., Donkor R., Adjei-Anang J., Ankamah-Lomotey S. (2021). Keratoconus in Africa: A Systematic Review and Meta-Analysis. Ophthalmic Physiol. Opt..

[9] Mittal P., Preethi B., Kumar K.K., Babu S.G., Srinivasa K.H. (2025). Epidemiology of Vernal Keratoconjunctivitis at a Tertiary Eye Care Centre in South India. Indian J. Clin. Exp. Ophthalmol..

[10] Fatima T., Acharya M.C., Mathur U., Barua P. (2010). Demographic Profile and Visual Rehabilitation of Patients with Keratoconus Attending Contact Lens Clinic at a Tertiary Eye Care Centre. Cont. Lens Anterior Eye.

[11] Shilpy N., Shah Z., Singh S., Purohit D. (2020). Prevalence of Keratoconus in Refractive Surgery Cases in Western India. Middle East. Afr. J. Ophthalmol..

[12] Umale R.H., Khan M.A., Moulick P.S., Gupta S., Shankar S., Sati A. (2019). A Clinical Study to Describe the Corneal Topographic Pattern and Estimation of the Prevalence of Keratoconus among Diagnosed Cases of Vernal Keratoconjunctivitis. Med. J. Armed Forces India.

[13] Sofi R.A., Mufti A. (2016). Vernal Keratoconjunctivitis in Kashmir: A Temperate Zone. Int. Ophthalmol..

[14] Saboo U.S., Jain M., Reddy J.C., Sangwan V.S. (2013). Demographic and Clinical Profile of Vernal Keratoconjunctivitis at a Tertiary Eye Care Center in India. Indian. J. Ophthalmol..

[15] Das A.V., Donthineni P.R., Sai Prashanthi G., Basu S. (2019). Allergic Eye Disease in Children and Adolescents Seeking Eye Care in India: Electronic Medical Records Driven Big Data Analytics Report II. Ocul. Surf..

[16] Nagachandrika T., Kumar U., Dumpati S., Chary S., Mandathara P.S., Rathi V.M. (2011). Prevalence of Contact Lens Related Complications in a Tertiary Eye Centre in India. Cont. Lens Anterior Eye.

[17] Das A.V., Deshmukh R.S., Reddy J.C., Joshi V.P., Singh V.M., Gogri P.Y., Murthy S.I., Chaurasia S., Fernandes M., Roy A. (2024). Keratoconus in India: Clinical Presentation and Demographic Distribution Based on Big Data Analytics. Indian. J. Ophthalmol..

[18] Jonas J.B., Nangia V., Matin A., Kulkarni M., Bhojwani K. (2009). Prevalence and Associations of Keratoconus in Rural Maharashtra in Central India: The Central India Eye and Medical Study. Am. J. Ophthalmol..

[19] Cleo G., Scott A.M., Islam F., Julien B., Beller E. (2019). Usability and Acceptability of Four Systematic Review Automation Software Packages: A Mixed Method Design. Syst. Rev..

[20] Lo C.K.-L., Mertz D., Loeb M. (2014). Newcastle-Ottawa Scale: Comparing Reviewers’ to Authors’ Assessments. BMC Med. Res. Methodol..

[21] Balshem H., Helfand M., Schünemann H.J., Oxman A.D., Kunz R., Brozek J., Vist G.E., Falck-Ytter Y., Meerpohl J., Norris S. (2011). GRADE Guidelines: 3. Rating the Quality of Evidence. J. Clin. Epidemiol..

[22] Guyatt G.H., Oxman A.D., Vist G.E., Kunz R., Falck-Ytter Y., Alonso-Coello P., Schünemann H.J. (2008). GRADE Working Group GRADE: An Emerging Consensus on Rating Quality of Evidence and Strength of Recommendations. BMJ.

[23] Higgins J.P.T., Thompson S.G. (2002). Quantifying Heterogeneity in a Meta-Analysis. Stat. Med..

[24] Barker T.H., Migliavaca C.B., Stein C., Colpani V., Falavigna M., Aromataris E., Munn Z. (2021). Conducting Proportional Meta-Analysis in Different Types of Systematic Reviews: A Guide for Synthesisers of Evidence. BMC Med. Res. Methodol..

[25] Wajnsztajn D., Solomon A. (2021). Vernal Keratoconjunctivitis and Keratoconus. Curr. Opin. Allergy Clin. Immunol..

[26] Naderan M., Rajabi M.T., Zarrinbakhsh P., Bakhshi A. (2017). Effect of Allergic Diseases on Keratoconus Severity. Ocul. Immunol. Inflamm..

[27] Merdler I., Hassidim A., Sorkin N., Shapira S., Gronovich Y., Korach Z. (2015). Keratoconus and Allergic Diseases among Israeli Adolescents between 2005 and 2013. Cornea.

[28] Dantas P.E.C., Alves M.R., Nishiwaki-Dantas M.C. (2005). Topographic Corneal Changes in Patients with Vernal Keratoconjunctivitis. Arq. Bras. Oftalmol..

[29] Gautam V., Chaudhary M., Sharma A.K., Shrestha G.S., Rai P.G. (2015). Topographic Corneal Changes in Children with Vernal Keratoconjunctivitis: A Report from Kathmandu, Nepal. Cont. Lens Anterior Eye.

[30] Cavas-Martínez F., De la Cruz Sánchez E., Nieto Martínez J., Fernández Cañavate F.J., Fernández-Pacheco D.G. (2016). Corneal Topography in Keratoconus: State of the Art. Eye Vis..

[31] Tachibana M., Adachi W., Kinoshita S., Kobayashi Y., Honma Y., Hiai H., Matsushima Y. (2002). Androgen-Dependent Hereditary Mouse Keratoconus: Linkage to an MHC Region. Invest. Ophthalmol. Vis. Sci..

[32] McMonnies C.W., Boneham G.C. (2003). Keratoconus, Allergy, Itch, Eye-Rubbing and Hand-Dominance. Clin. Exp. Optom..

[33] Hashemi H., Beiranvand A., Khabazkhoob M., Asgari S., Emamian M.H., Shariati M., Fotouhi A. (2013). Prevalence of Keratoconus in a Population-Based Study in Shahroud. Cornea.

[34] Fink B.A., Wagner H., Steger-May K., Rosenstiel C., Roediger T., McMahon T.T., Gordon M.O., Zadnik K. (2005). Differences in Keratoconus as a Function of Gender. Am. J. Ophthalmol..

[35] Moher D., Liberati A., Tetzlaff J., Altman D.G., The PRISMA Group (2009). Preferred Reporting Items for Systematic Reviews and Meta-Analyses: The PRISMA Statement. PLoS Med..

