# Supplementary material for: Epidemiology of Keratoconus in India: A Systematic Review and Meta-Analysis of Indian Study Populations
Source: Vision (Basel). 2026 Apr 9;10(2):20. doi: 10.3390/vision10020020 (PMC13108012; doi:10.3390/vision10020020)
Supplement: Supplementary file 1 [file vision-10-00020-s001.zip › Supplementary Material S1.pdf]

Detailed Search Strategy

PUBMED:
Free Text and MESH
Search updated on 8 June 2025

Query: Search: (((frequency”[All Fields]) OR (prevalence”[All Fields])) OR (incidence”[All Fields])) AND (((India) OR ((((((((((((((((((((((((((((Andhra Pradesh) OR (Arunachal Pradesh)) OR (Assam)) OR (Bihar)) OR (Chhattisgarh)) OR (Goa)) OR (Gujarat)) OR (Haryana)) OR (Himachal Pradesh)) OR (Jharkhand)) OR (Karnataka)) OR (Kerala)) OR (Madhya Pradesh)) OR (Maharashtra)) OR (Manipur)) OR (Meghalaya)) OR (Mizoram)) OR (Nagaland)) OR (Odisha)) OR (Punjab)) OR (Rajasthan)) OR (Sikkim)) OR (Tamil Nadu)) OR (Telangana)) OR (Tripura)) OR (Uttar Pradesh)) OR (Uttarakhand)) OR (West Bengal))) AND (keratoconus”[All Fields])) Sort by: Most Recent
("frequency"“[All Fields] OR "prevalence"“[All Fields] OR "incidence"“[All Fields]) AND (("india"[MeSH Terms] OR "india"[All Fields] OR "india s"[All Fields] OR "indias"[All Fields] OR (pradesh, andhra[Author] OR pradesh, andhra[Investigator] OR ("Arunachal"[All Fields] AND "Pradesh"[All Fields]) OR "Assam"[All Fields] OR "Bihar"[All Fields] OR "Chhattisgarh"[All Fields] OR "Goa"[All Fields] OR "Gujarat"[All Fields] OR "Haryana"[All Fields] OR ("Himachal"[All Fields] AND "Pradesh"[All Fields]) OR "Jharkhand"[All Fields] OR "Karnataka"[All Fields] OR "Kerala"[All Fields] OR pradesh, madhya[Investigator] OR "Maharashtra"[All Fields] OR "Manipur"[All Fields] OR "Meghalaya"[All Fields] OR "Mizoram"[All Fields] OR "Nagaland"[All Fields] OR "Odisha"[All Fields] OR "Punjab"[All Fields] OR "Rajasthan"[All Fields] OR ("sikkim"[MeSH Terms] OR "sikkim"[All Fields]) OR nadu, tamil[Investigator] OR "Telangana"[All Fields] OR "Tripura"[All Fields] OR ("Uttar"[All Fields] AND "Pradesh"[All Fields]) OR "Uttarakhand"[All Fields] OR ("West"[All Fields] AND ("bengal"[All Fields] OR "bengale"[All Fields] OR "bengals"[All Fields])))) AND "keratoconus"“[All Fields])

#	**Search Term**	**No. Results**
1	"India"	835.111
2	"Andhra Pradesh” OR “Arunachal Pradesh” OR “Assam” OR “Bihar” OR “Chhattisgarh” OR “Goa” OR “Gujarat” OR “Haryana” OR “Himachal Pradesh” OR “Jharkhand” OR “Karnataka” OR “Kerala” OR “Madhya Pradesh” OR “Maharashtra” OR “Manipur” OR “Meghalaya” OR “Mizoram” OR “Nagaland” OR “Odisha” OR “Punjab” OR “Rajasthan” OR “Sikkim” OR “Tamil Nadu” OR “Telangana” OR “Tripura” OR “Uttar Pradesh” OR “Uttarakhand” OR “West Bengal"	285.350
4	1 OR 2	851.901
5	“Keratoconus”	9.559
6	“Prevalence” OR “Frequency” OR “Incidence”	2.627.218
7	4 AND 5 AND 6	32

EMBASE

Free Text
Search updated on 8 June 2025

Mapping options enabled:

- map to preferred term in Emtree
- search also as free text in all fields
- explode using narrower Emtree terms
- search as broadly as possible

#	Search Term	No. of Results
1	(India) OR (Andhra AND Pradesh) OR (Arunachal AND Pradesh) OR Assam OR Bihar OR Chhattisgarh OR Goa OR Gujarat OR Haryana OR 'Himachal pradesh' OR Jharkhand OR Karnataka OR Kerala OR 'Madhya Pradesh' OR Maharashtra OR Manipur OR Meghalaya OR Mizoram OR Nagaland OR Odisha OR Punjab OR Rajasthan OR Sikkim OR (Tamil AND Nadu) OR Telangana OR Rripura OR (Uttar AND Pradesh) OR Uttarakhand OR (West AND Bengal)) AND Keratoconus AND “Prevalence” OR “Frequency” OR “Incidence”	39

SCOPUS

Free Text
Search updated on 8 June 2025

#	Search Term	No. of Results
1	(TITLE-ABS-KEY (andhra AND pradesh) OR TITLE-ABS-KEY (arunachal AND pradesh) OR TITLE-ABS-KEY (assam) OR TITLE-ABS-KEY (bihar) OR TITLE-ABS-KEY (chhattisgarh) OR TITLE-ABS-KEY (goa) OR TITLE-ABS-KEY (gujarat) OR TITLE-ABS-KEY (haryana) OR TITLE-ABS-KEY (himachal AND pradesh) OR TITLE-ABS-KEY (jharkhand) OR TITLE-ABS-KEY (karnataka) OR TITLE-ABS-KEY (kerala) OR TITLE-ABS-KEY (madhya AND pradesh) OR TITLE-ABS-KEY (maharashtra) OR TITLE-ABS-KEY (manipur) OR TITLE-ABS-KEY (meghalaya) OR TITLE-ABS-KEY (mizoram) OR TITLE-ABS-KEY (nagaland) OR TITLE-ABS-KEY (odisha) OR TITLE-ABS-KEY (punjab) OR TITLE-ABS-KEY (rajasthan) OR TITLE-ABS-KEY (sikkim) OR TITLE-ABS-KEY (tamil AND nadu) OR TITLE-ABS-KEY (telangana) OR TITLE-ABS-KEY (tripura) OR TITLE-ABS-KEY (uttar AND pradesh) OR TITLE-ABS-KEY (uttarakhand) OR TITLE-ABS-KEY (west AND bengal) OR TITLE-ABS-KEY (india) AND TITLE-ABS-KEY (keratoconus) AND TITLE-ABS-KEY (frequency) OR TITLE-ABS-KEY (incidence) OR TITLE-ABS-KEY (prevalence))	30
