# Supplementary material for: Epidemiology of Keratoconus in India: A Systematic Review and Meta-Analysis of Indian Study Populations
Source: Vision (Basel). 2026 Apr 9;10(2):20. doi: 10.3390/vision10020020 (PMC13108012; doi:10.3390/vision10020020)
Supplement: Supplementary file 1 [file vision-10-00020-s001.zip › Supplementary Material S3.pdf]

Table S1: Characteristics of studies included in the systematic review

First Author	Year	State	Study Design	Study Population	Age Range	Total Sample Size (n.)<br><br>Number of Patients with KC (n. and %)<br><br>Number of Eyes with KC (n. and %)	Age of KC patients (years or range)<br><br>Gender (n. and %)	Site (Monolateral Vs Bilateral, n. and %)	Diagnostic Test Used	Risk Factors and/or Ocular co-morbidities	KC Stage	KC Definition and/or Grading
Jonas et al. [18]	2009	Maharashtra	Cross-Sectional Study	Rural Population (Screening Camps)	Adults: >30 years	4667 Patients<br><br>128 (2.74%)<br><br>212 (4.54%)	53.2 ± 11.3<br><br>Male: 29 (22.65%)<br>Female: 99 (77.35%)	N/a	SL examination<br>Keratometry	N/a	N/a	Mean spherical equivalent of the anterior corneal refractive power of ≥ 48 diopters
Nagachandrika et al. [16]	2011	Telangana	Cross-Sectional Study	Contact-Lens Wearer Patients	Adults: >18 years	1089 patients<br><br>159 (14.6%)<br><br>N/a	N/a<br><br>N/a	N/a<br><br>N/a	N/a<br><br>N/a	N/a<br><br>N/a	N/a	N/a<br><br>N/a
Saboo, et al. [14]	2013	Telangana	Cross-Sectional Study	VKC	Pediatric	468 patients<br><br>29 (6.19%)<br><br>N/a	N/a<br><br>N/a	N/a<br><br>N/a	SL examination<br>Corneal Topography	VKC	N/a	N/a<br><br>N/a
Sofi et al. [13]	2016	Jammu and Kashmir	Cross-Sectional Study	VKC	0-25	212 patients<br><br>13 (6%)<br><br>N/a	N/a<br><br>N/a	N/a<br><br>N/a	N/a<br><br>N/a	VKC	N/a	N/a<br><br>N/a

First Author	Year	State	Study Design	Study Population	Age Range	Total Sample Size (n.)<br><br>Number of Patients with KC (n. and %)<br><br>Number of Eyes with KC (n. and %)	Age of KC patients (years or range)<br><br>Gender (n. and %)	Site (Monolateral Vs Bilateral, n. and %)	Diagnostic Test Used	Risk Factors and/or Ocular co-morbidities	KC Stage	KC Definition and/or Grading
Umale et al. [12]	2018	Maharashtra	Cross-Sectional Study	VKC	10 ≤ age ≤ 30 years	152 eyes of the 76 patients<br><br>11 (7.2%)<br><br>17 (11.18%)	10-15: 8 (10.26%)<br>16-20: 7 (13.46%)<br>21-25: 0<br>26-30: 2 (25%)<br><br>Male: 8 (47.05%)<br>Female: 9 (52.95%)	Monolateral: 5 (45.45%)<br>Bilateral: 6 (54.54%)	SL examination<br>Corneal Topography	VKC	N/a	RabinowitzeMcDonnell criteria of KC
Das et al. [15]	2019	Telangana<br>Andhra Pradesh<br>Odisha<br>Karnataka	Cross-Sectional Study	AED	≤21 years of age	51.917 eyes of the 26.309 patients<br><br>708 (1.36%)<br><br>N/a	N/a<br><br>N/a	N/a<br><br>N/a	N/a<br><br>N/a	AED	N/a	N/a<br><br>N/a
Shilpy et al. [11]	2020	Gujarat	Cross-Sectional Study	RS	18 ≤ age ≤ 40 years	2902 patients<br><br>47 (1.62%)<br><br>N/a	24.62 ±5.37<br><br>Male: 17 (36.17%)<br>Female: 30 (63.83%)	N/a	N/a	N/a	N/a	N/a
Mittal et al. [9]	2022	Karnataka	Cross-Sectional Study	VKC	0 ≤ age ≤ 20 years	1684 patients<br><br>243 (14.42%)<br><br>N/a	N/a<br><br>N/a	N/a<br><br>N/a	N/a<br><br>N/a	VKC	N/a	N/a

First Author	Year	State	Study Design	Study Population	Age Range	Total Sample Size (n.)<br><br>Number of Patients with KC (n. and %)<br><br>Number of Eyes with KC (n. and %)	Age of KC patients (years or range)<br><br>Gender (n. and %)	Site (Monolateral Vs Bilateral, n. and %)	Diagnostic Test Used	Risk Factors and/or Ocular co-morbiditie s	KC Stage	KC Definition and/or Grading
Fatima et al. [10]	2022	Delhi	Cross-Sectional Study	Contact-Lens Wearer Patients	24 (15–52)	856 patients<br><br>77 (8.99%)<br><br>142 (16.58%)	24 (15–36)<br><br>Male: 49 (63%)<br>Female: 28 (37%)	N/a	SL examination Corneal Topography	N/a	Mild: 20 eyes (14.4%)<br>Moderate: 51 eyes (36.7%)<br>Advanced: 45(32.4%)<br>Severe: 23 eyes (16.6%)	Grading based on keratometry was as follows:<br>Mild KC <45 D in both meridians<br>Moderate KC 45–52 D in 1 or both meridians<br>Advanced KC >52 D in 1 or both meridians<br>Severe KC >62 D in 1 or both meridians
Das et al. [17]	2024	Telangana<br>Andhra Pradesh<br>Odisha<br>Karnataka	Cross-Sectional Study	Electronic medical records of all patients	Adults and Pediatrics	2.384.523 patients<br><br>14.749 (0.62%)<br><br>27.703 (0.58%)	22.61 ± 8.23<br><br>Male: 9.034 (61.36%)<br>Female: 5.715 (38.75%)	Monolateral: 12.954 (87.83%)<br>Bilateral: 1.795 (12.17%)	SL examination Corneal Topography	VKC<br>AED<br><br>Socioeconomic Status<br><br>Geography Status	Stage 1: 3.186 (50.64%)<br><br>Stage 2: 1.703 (27.07%)<br><br>Stage 3: 370 (5.88%)<br><br>Stage 4: 1.033 (16.42%)	Amsler Krumeich

Abbreviations: N/A: not applicable; n. number; KC: Keratoconus; SL: Slit Lamp; D: diopters; VKC: Vernal Keratoconjunctivitis; AED: Allergic Eye Disease; RS: Refractive Surgery; D: Diopters.

Table S2: Meta-analyses of subgroups

	Studies	Prevalence of KC (95% CI)	P	I^2
**KC Prevalence**				
**Geographic Region**				
North	2	0.08 (0.05, 0.11)	0.14	55.52
West	3	0.05 (0.02, 0.12)	<0.001	99.96
South	5	0.07 (0.01, 0.13)	<0.001	99.99
**KC Assessment**				
CT	4	0.09 (0.03, 0.16)	<0.001	98.24
Keratometry	1	0.03 (0.02, 0.03)	/	/
Not Reported	5	0.06 (0.01, 0.11)	<0.001	99.64
**Study Population**				
AED	1	0.01 (0.01, 0.01)	/	/
VKC	2	0.10 (0.05, 0.15)	<0.001	91.19
CL Wearers	2	0.12 (0.06, 0.17)	<0.001	93.31
Refractive Surgery Candidates	1	0.02 (0.01, 0.02)	/	/
General Population	2	0.02 (0.00, 0.04)	<0.001	78.91

Abbreviations: KC: Keratoconus, CI: Confidence Interval, I^2: Heterogeneity, CT: Corneal Topography; AED: Allergic Eye Disease, VKC: Vernal Keratoconjunctivitis, CL: Contact Lens, I: Heterogeneity
P and I^2 for subgroups with only 1 constituent study are not reported and instead indicated with a Slash (/).
We urge readers to exercise caution in interpreting these exploratory analyses, especially for subgroups with few constituent studies.

Table S3: Newcastle–Ottawa Scale for Critical Appraisal of Cross-Sectional Studies

		Selection				Comparability	Outcome		
**Source**	**Study design**	**Representativeness of the sample**	**Sample size**	**Non-respondents**	**Ascertainment of the exposure:**		**Assessment of outcome**	**Statistical test**	**Total score**
Jonas et al., 2009 [18]	Cross-Sectional	+1 (a)	+1 (a)	+1 (b)	+1 (b)	+2 (a)	+1 (c)	+1 (a)	8/10
Nagachandrika et al., 2011 [16]	Cross-Sectional	+1 (b)	+1 (a)	+1 (b)	0 (c)	0 (b)	+2 (b)	+1 (a)	7/10
Saboo, et al., 2013 [14]	Cross-Sectional	+1 (b)	+1 (a)	+1 (b)	0 (c)	0 (b)	+2 (b)	+1 (a)	6/10
Sofi et al., 2016 [13]	Cross-Sectional	+1 (b)	0 (b)	+1 (b)	0 (c)	+1 (a)	+2 (b)	+1 (a)	6/10
Das et al., 2009 [15]	Cross-Sectional	+1 (b)	+1 (a)	+1 (b)	0 (c)	+2 (a)	+2 (b)	+1 (a)	8/10
Umale et al., 2018 [12]	Cross-Sectional	+1 (b)	0 (b)	+1 (b)	+2 (a)	+2 (a)	+2 (a)	+1 (a)	9/10
Shilpy et al., 2020 [11]	Cross-Sectional	+1 (b)	+1 (a)	+1 (b)	0 (c)	0 (b)	+2 (b)	+1 (a)	6/10
Mittal et al., 2022 [9]	Cross-Sectional	+1 (b)	+1 (a)	+1 (b)	0 (c)	0 (b)	+2 (b)	+1 (a)	6/10
Fatima et al., 2022 [10]	Cross-Sectional	+1 (b)	+1 (a)	+1 (b)	0 (c)	0 (b)	+2 (b)	+1 (a)	6/10
Das et al., 2024 [17]	Cross-Sectional	+1 (a)	+1 (a)	+1 (b)	+2 (a	+2 (a)	+2 (a)	+1 (a)	10/10

Representativeness of the sample: a) Truly representative of the average in the target population. * b) Somewhat representative of the average in the target population; * c) Selected group of users d) No description of the sampling strategy; **Sample size**: a) Justified and satisfactory. * b) Not justified; **Non-respondents**: a) Comparability between respondents and non-respondents characteristics is established, and the response rate is satisfactory. * b) The response rate is unsatisfactory, or the comparability between respondents and nonrespondents is unsatisfactory. c) No description of the response rate or the characteristics of the responders and the nonresponders.; Ascertainment of the exposure: a) Validated measurement tool. * b) Non-validated measurement tool, but the tool is available or described. c) No description of the measurement tool.; **Comparability: (Maximum 2 stars)** 1) The subjects in different outcome groups are comparable, based on the study design or analysis. Confounding factors are controlled. a) Data/ results adjusted for relevant predictors/risk factors/confounders ** b. Data/results not adjusted for all relevant confounders/risk factors/information not provided. **Outcome (Maximum 2 stars):** 1) Assessment of the outcome: a) Independent blind assessment *; b) Record linkage *; c) Self report; d) No description. 2) Statistical test: a) The statistical test used to analyze the data is clearly described and appropriate, and the measurement of the association is presented, including confidence intervals and the probability level (p value) *; b) The statistical test is not appropriate, not described or incomplete.
Cross-sectional Studies: Very Good Studies: 9-10 points Good Studies: 7-8 points Satisfactory Studies: 5-6 points Unsatisfactory Studies: 0 to 4 points

Table S4: GRADE assessment

Certainty assessment									Certainty
**Outcome**	**Studies (n)**	**Study design**	**Risk of bias**	**Inconsistency[a]**	**Indirectness[d]**	**Imprecision[c]**	**Publication bias[b]**	**Patients (n)**	
Prevalence of Keratoconus	10	Cross-sectional Studies	serious	Very serious	Serious	Not serious	/	16.164	⊕◯◯◯ Very Low
Prevalence of Keratoconus according to Gender	6	Cross-sectional Studies		Very serious	Serious	Not serious	/	15720	⊕◯◯◯ Very Low
Prevalence of Keratoconus according Laterality	2	Cross-sectional Studies		Very serious	Serious	Not serious	/	14760	⊕◯◯◯ Very Low

CI: Confidence interval; **MD:** Mean difference; **OR:** Odds ratio; [a] Substantial heterogeneity I^2 > 60% (serious) or >90% (very serious); [b] Strongly suspected if funnel plot suggestive of publication bias or lack of small studies and negative effects; [c] serious if total number of events is less than 300, CIs overlap or non clinically significant effect; [d] Serious indirectness refer to variation of outcome measure or definition across studies

References:

9. Mittal, P.; Preethi, B.; Kumar, K.K.; Babu, S.G.; Srinivasa, K.H. Epidemiology of Vernal Keratoconjunctivitis at a Tertiary Eye Care Centre in South India. *Indian J. Clin. Exp. Ophthalmol.* **2025**, *8*, 217–220. https://doi.org/10.18231/j.ijceo.2022.043.
10. Fatima, T.; Acharya, M.C.; Mathur, U.; Barua, P. Demographic Profile and Visual Rehabilitation of Patients with Keratoconus Attending Contact Lens Clinic at a Tertiary Eye Care Centre. *Cont. Lens Anterior Eye* **2010**, *33*, 19–22. https://doi.org/10.1016/j.clae.2009.09.004.
11. Shilpy, N.; Shah, Z.; Singh, S.; Purohit, D. Prevalence of Keratoconus in Refractive Surgery Cases in Western India. *Middle East. Afr. J. Ophthalmol.* **2020**, *27*, 156–159. https://doi.org/10.4103/meajo.MEAJO_182_19.
12. Umale, R.H.; Khan, M.A.; Moulick, P.S.; Gupta, S.; Shankar, S.; Sati, A. A Clinical Study to Describe the Corneal Topographic Pattern and Estimation of the Prevalence of Keratoconus among Diagnosed Cases of Vernal Keratoconjunctivitis. *Med. J. Armed Forces India* **2019**, *75*, 424–428. https://doi.org/10.1016/j.mjafi.2018.07.012.
13. Sofi, R.A.; Mufti, A. Vernal Keratoconjunctivitis in Kashmir: A Temperate Zone. *Int. Ophthalmol.* **2016**, *36*, 875–879. https://doi.org/10.1007/s10792-016-0213-8.
14. Saboo, U.S.; Jain, M.; Reddy, J.C.; Sangwan, V.S. Demographic and Clinical Profile of Vernal Keratoconjunctivitis at a Tertiary Eye Care Center in India. *Indian. J. Ophthalmol.* **2013**, *61*, 486–489. https://doi.org/10.4103/0301-4738.119431.
15. Das, A.V.; Donthineni, P.R.; Sai Prashanthi, G.; Basu, S. Allergic Eye Disease in Children and Adolescents Seeking Eye Care in India: Electronic Medical Records Driven Big Data Analytics Report II. *Ocul. Surf.* **2019**, *17*, 683–689. https://doi.org/10.1016/j.jtos.2019.08.011.
16. Nagachandrika, T.; Kumar, U.; Dumpati, S.; Chary, S.; Mandathara, P.S.; Rathi, V.M. Prevalence of Contact Lens Related Complications in a Tertiary Eye Centre in India. *Cont. Lens Anterior Eye* **2011**, *34*, 266–268. https://doi.org/10.1016/j.clae.2011.06.008.
17. Das, A.V.; Deshmukh, R.S.; Reddy, J.C.; Joshi, V.P.; Singh, V.M.; Gogri, P.Y.; Murthy, S.I.; Chaurasia, S.; Fernandes, M.; Roy, A.; et al. Keratoconus in India: Clinical Presentation and Demographic Distribution Based on Big Data Analytics. *Indian. J. Ophthalmol.* **2024**, *72*, 105–110. https://doi.org/10.4103/IJO.IJO_1190_23.
18. Jonas, J.B.; Nangia, V.; Matin, A.; Kulkarni, M.; Bhojwani, K. Prevalence and Associations of Keratoconus in Rural Maharashtra in Central India: The Central India Eye and Medical Study. *Am. J. Ophthalmol.* **2009**, *148*, 760–765. https://doi.org/10.1016/j.ajo.2009.06.024.
