# Supplementary material for: Epidemiology of Keratoconus in India: A Systematic Review and Meta-Analysis of Indian Study Populations
Source: Vision (Basel). 2026 Apr 9;10(2):20. doi: 10.3390/vision10020020 (PMC13108012; doi:10.3390/vision10020020)
Supplement: Supplementary file 1 [file vision-10-00020-s001.zip › Supplementary Material S4 .pdf]

Figure S1: Analysis of heterogeneity across studies: Galbraith plot

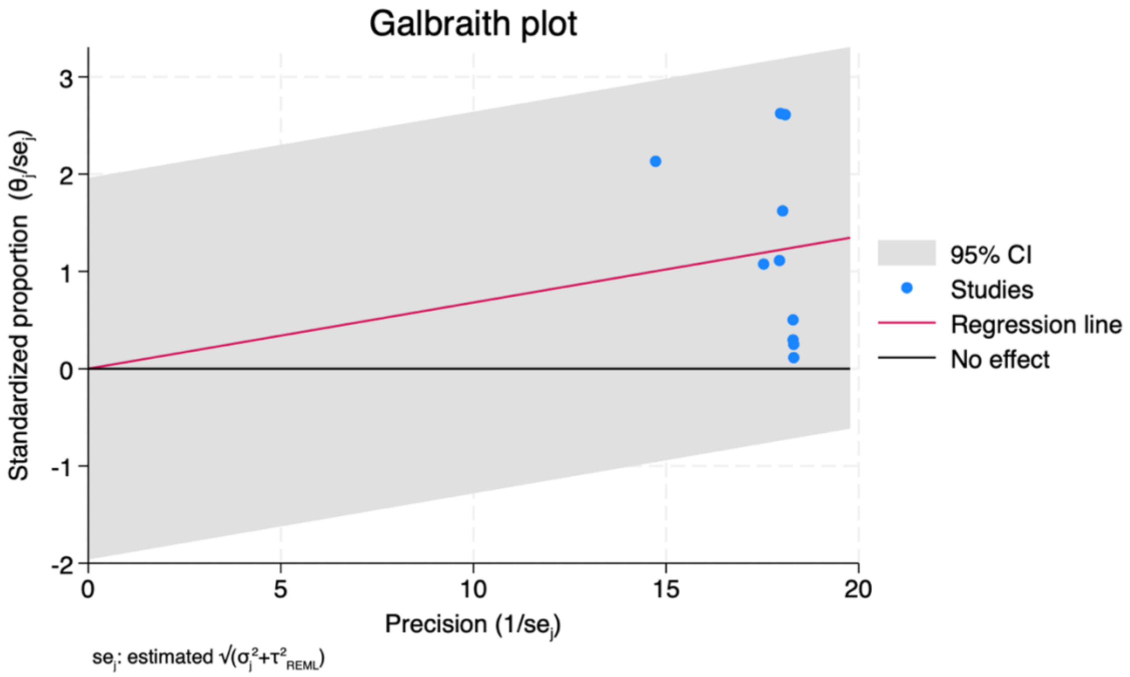

Figure S1. Galbraith plot for analysis of heterogeneity across studies. The red line depicted the regression line, parallel to the regression line, at a 2-standard-deviation distance, 2 lines (dotted green lines) created an interval in which all 10 studies (in small circles) fell, indicating no inconsistency was found across studies.

Figure S2: Analysis of heterogeneity across studies: leave-one-out method

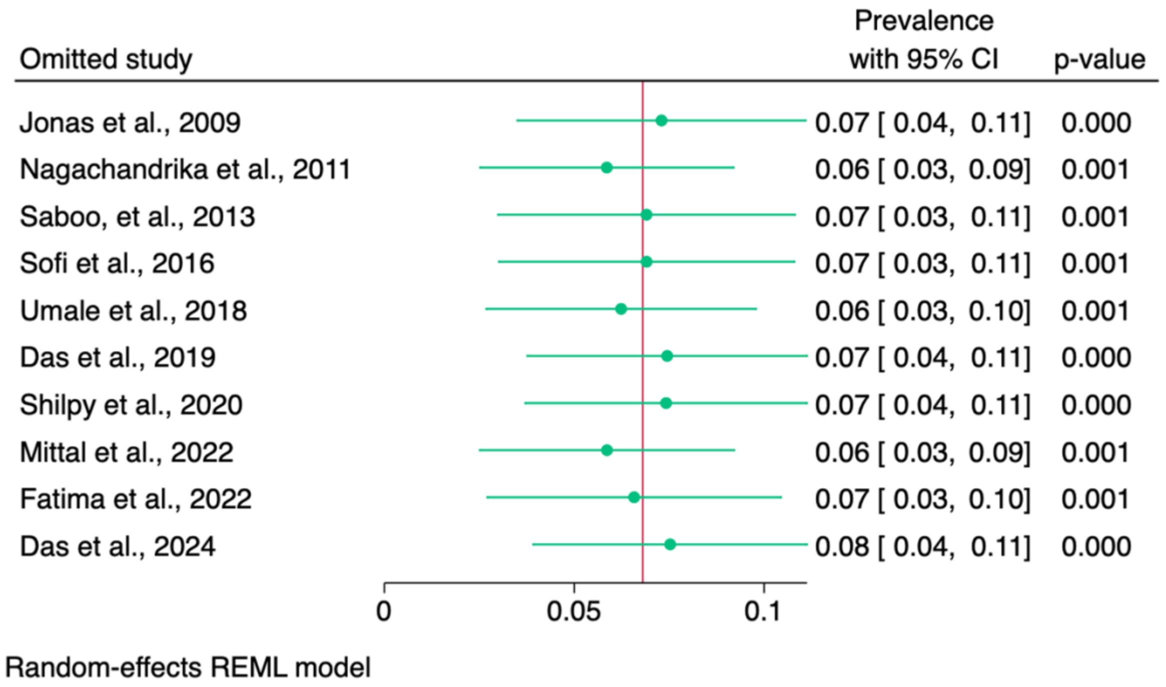

Figure S2. Analysis of heterogeneity across studies: leave-one-out method. The results of the leave-one-out method in sensitivity analysis. The horizontal lines and circles indicate the prevalence and 95% Confidence Intervals applying the leave-one-out method.

Figure S3: Random-effects meta-analyses of the Keratoconus Prevalence: Geographic region

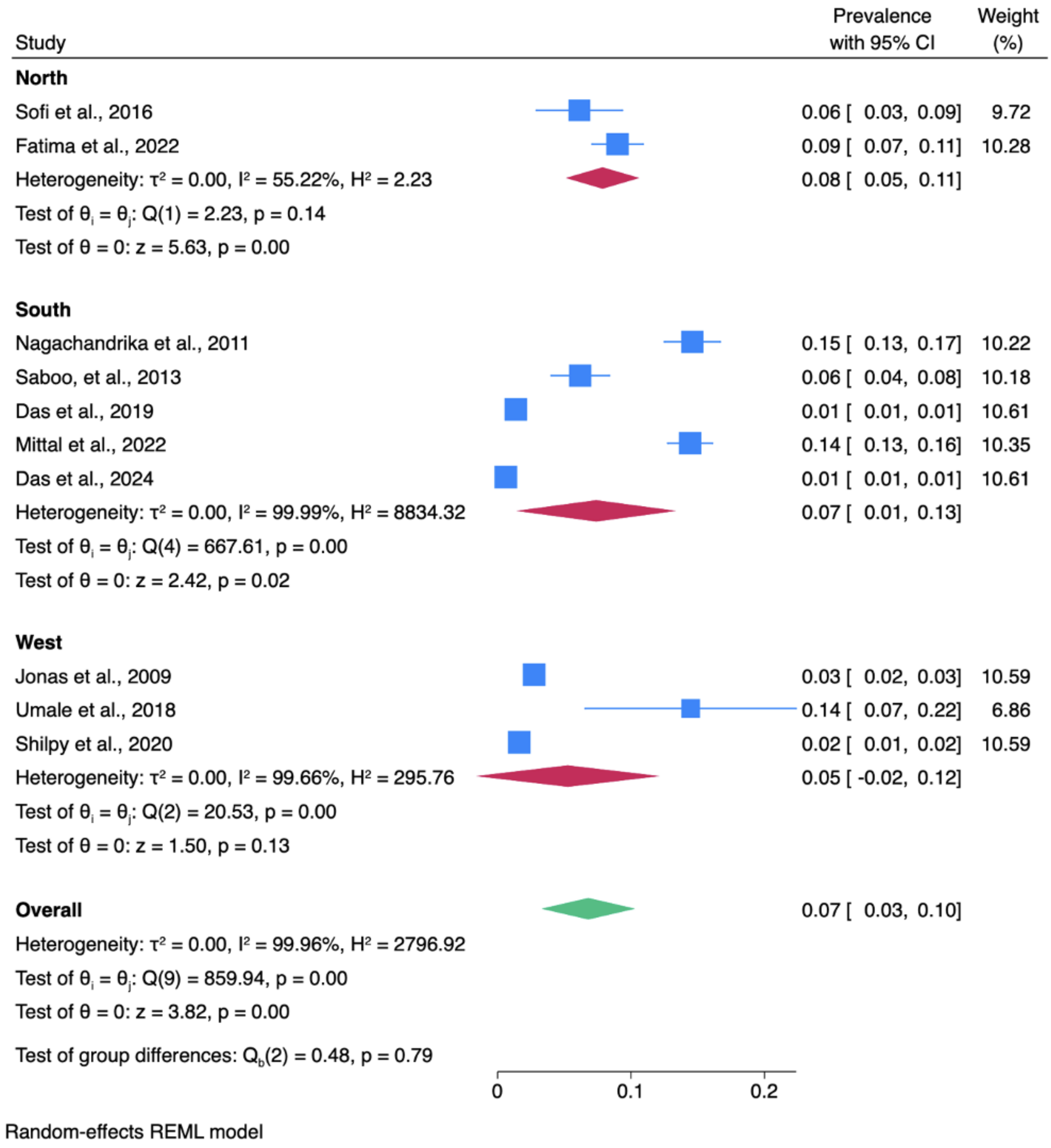

Figure S3. Random-effects meta-analyses of the Keratoconus Prevalence: Geographic region. Subgroup analyses of studies according to the geographic region where the data research was retrieved (studies were divided into North, West, and South subgroups).

Figure S4: Random-effects meta-analyses of the Keratoconus Prevalence: KC Assessment

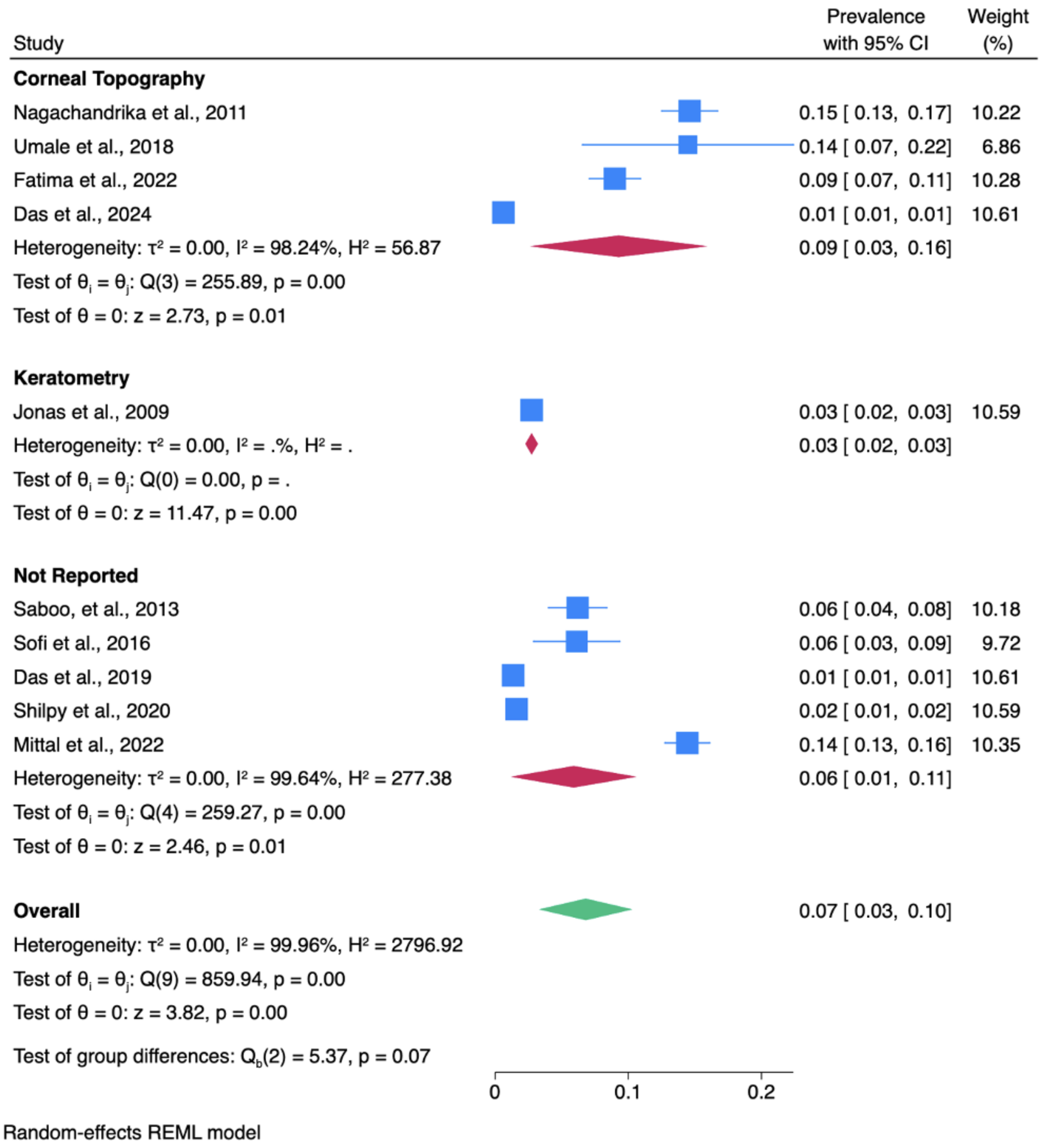

Figure S4. Random-effects meta-analyses of the Keratoconus Prevalence: KC Assessment. Subgroup analyses of studies according to the Keratoconus assessment (studies were divided into Corneal Topography, Not Reported, and Keratometry).

Figure S5: Random-effects meta-analyses of the Keratoconus Prevalence: Gender

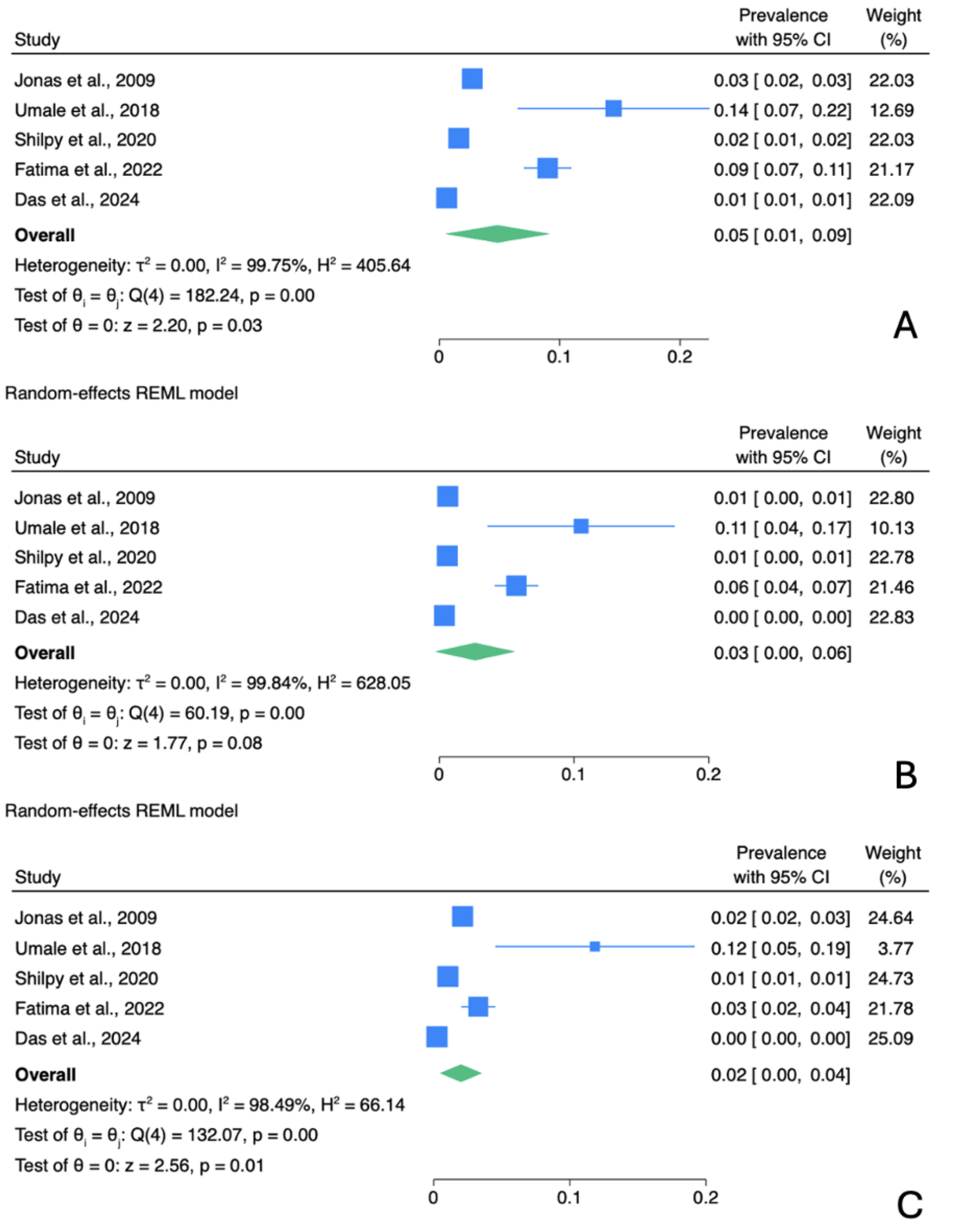

Figure S5. Random-effects meta-analyses of the Keratoconus Prevalence: Gender. Forest plot of the prevalence of keratoconus by gender. (A: Prevalence of Keratoconus in the Total Population included in the 5 retrieved studies, B: Prevalence of Keratoconus in males, C: Prevalence of Keratoconus in females).

Figure S6: Random-effects meta-analyses of the Keratoconus Prevalence: Laterality

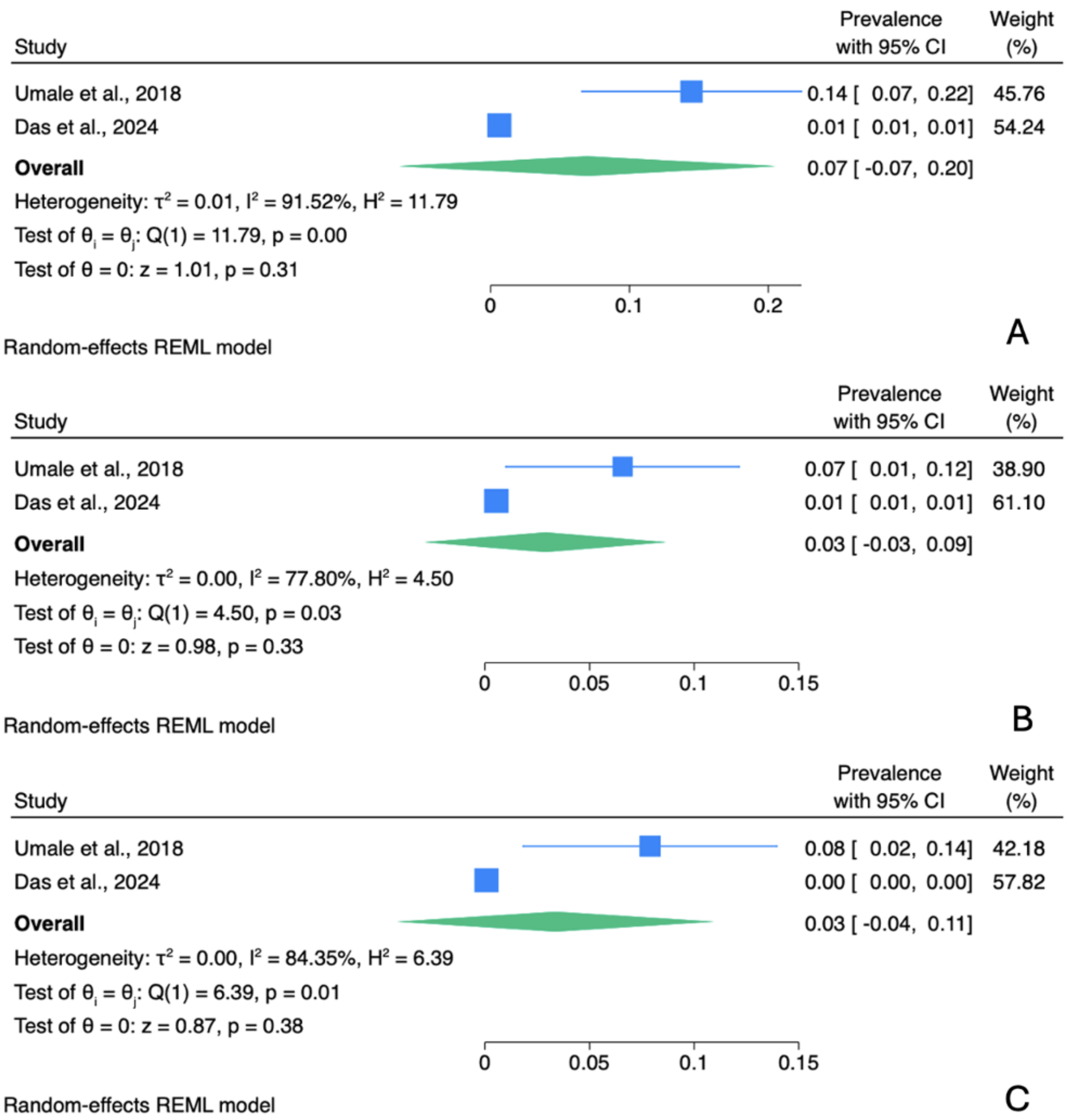

Figure S6. Random-effects meta-analyses of the Keratoconus Prevalence: Laterality. Forest plot of the prevalence of keratoconus by Laterality. (A: Prevalence of Keratoconus in the Total Population included in the 2 retrieved studies, B: Prevalence of Keratoconus in Monolateral, C: Prevalence of Keratoconus in Bilateral).
